# Supplementary figures and images for: Dock1 functions in Schwann cells to regulate development, maintenance, and repair
Source: J Cell Biol. 2025 Mar 19;224(5):e202311041. doi: 10.1083/jcb.202311041 (PMC11921805; doi:10.1083/jcb.202311041)

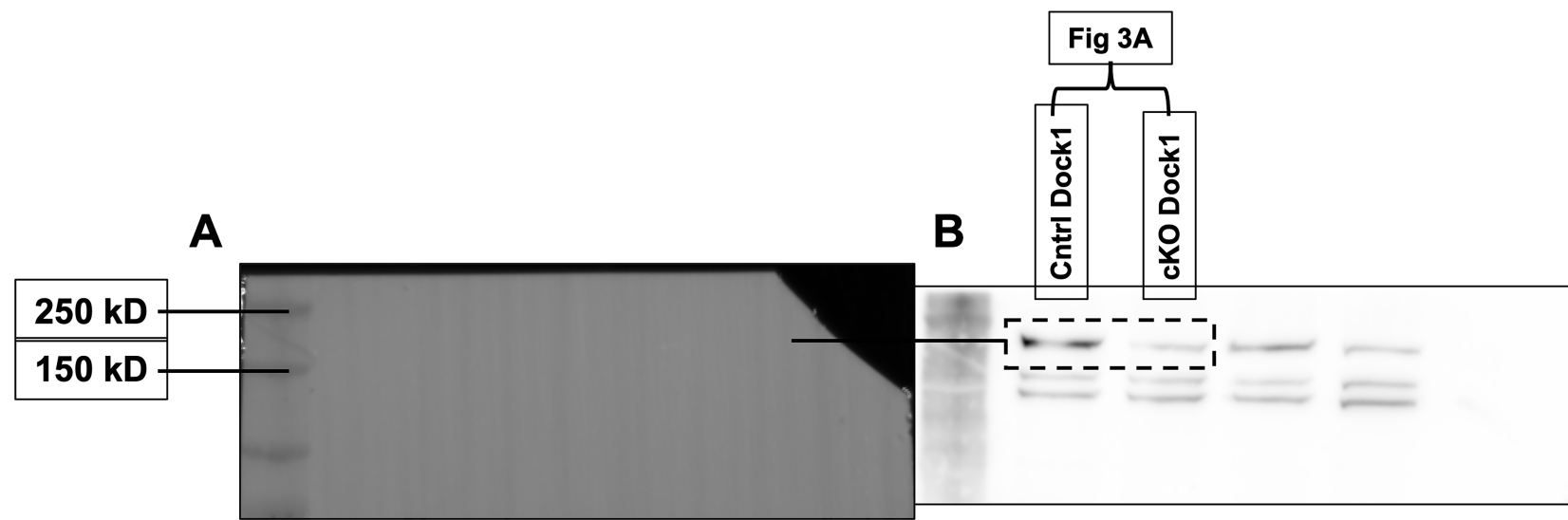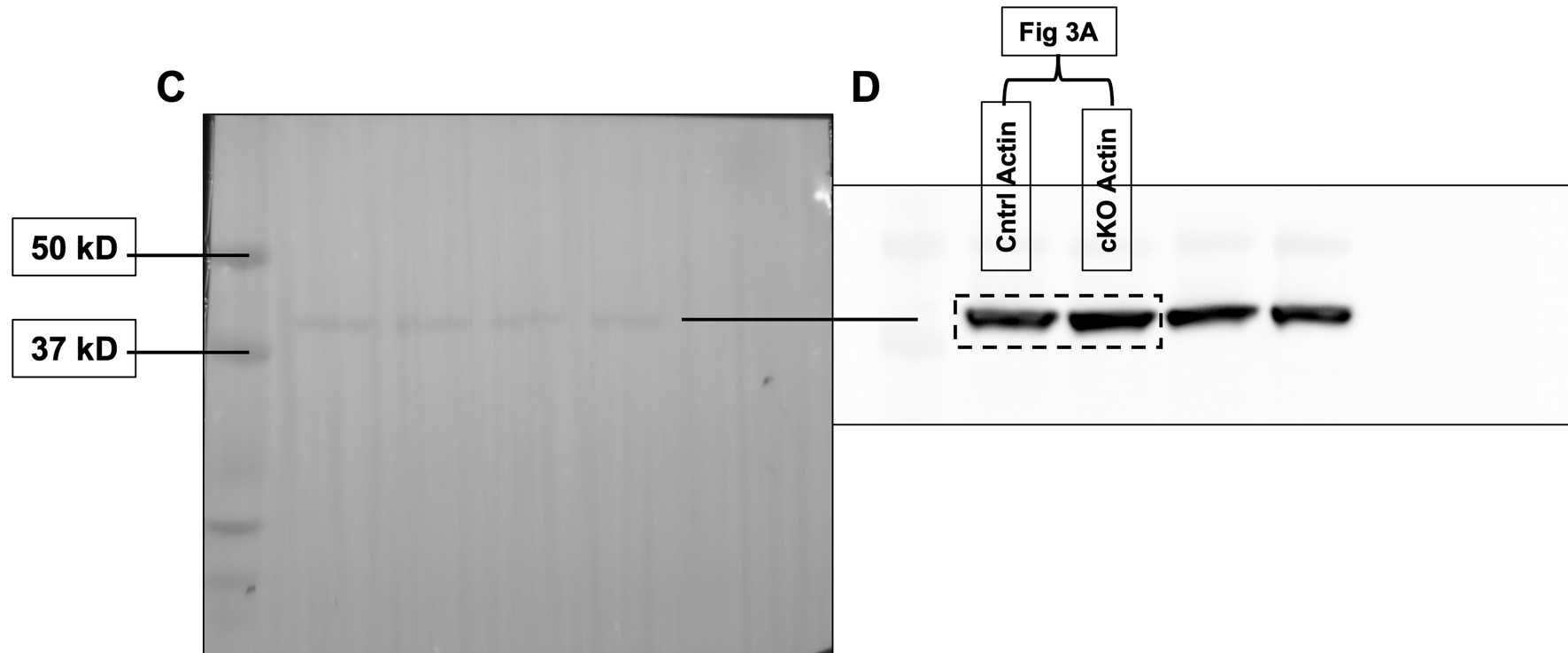

Supplement: SourceData F3 — is the source file for Fig. 3. [file jcb_202311041_sourcedataf3.pdf]

**A**

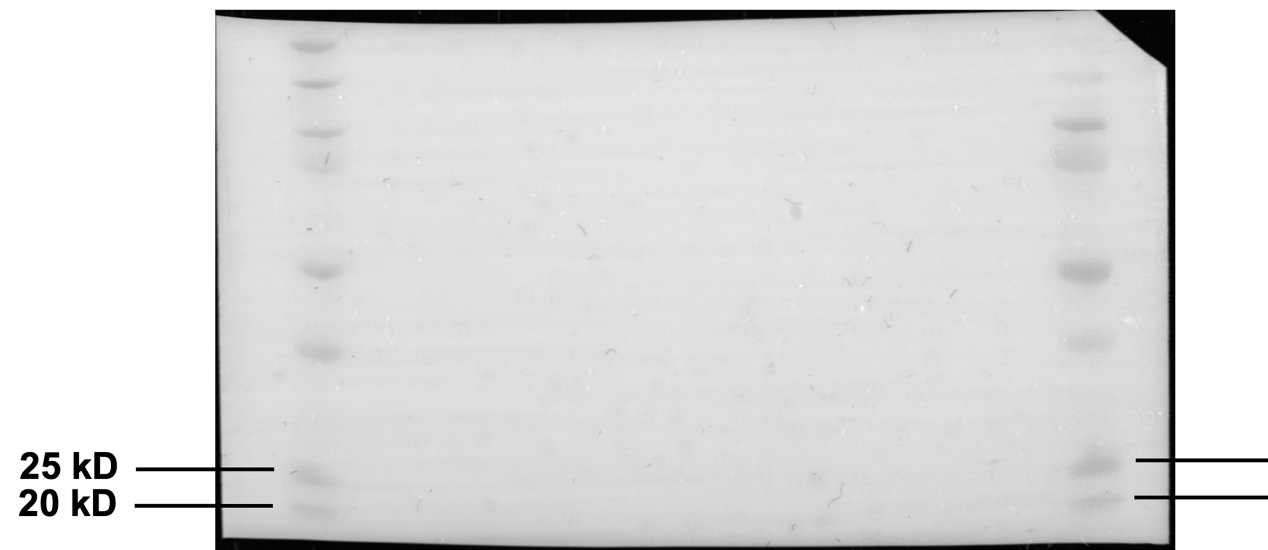

**B**

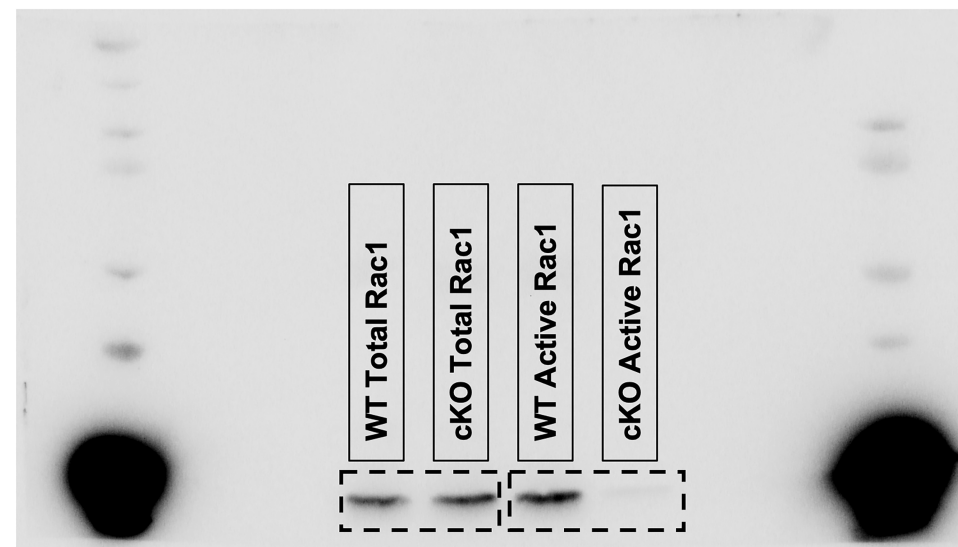

Supplement: SourceData F7 — is the source file for Fig. 7. [file jcb_202311041_sourcedataf7.pdf]

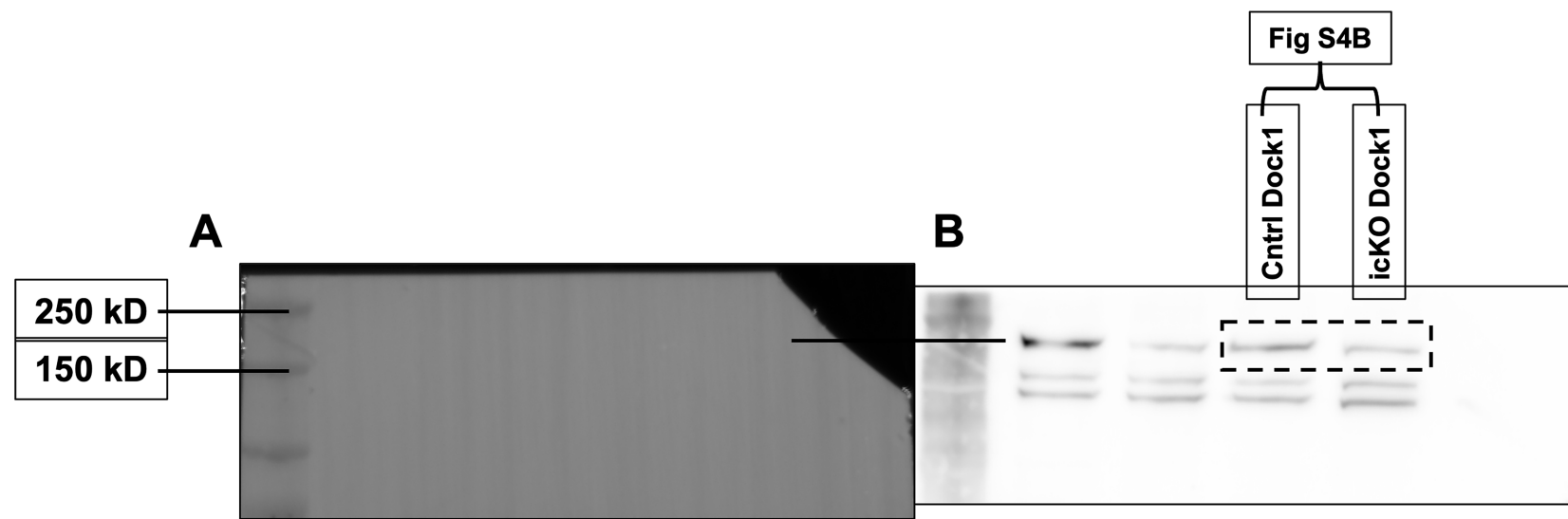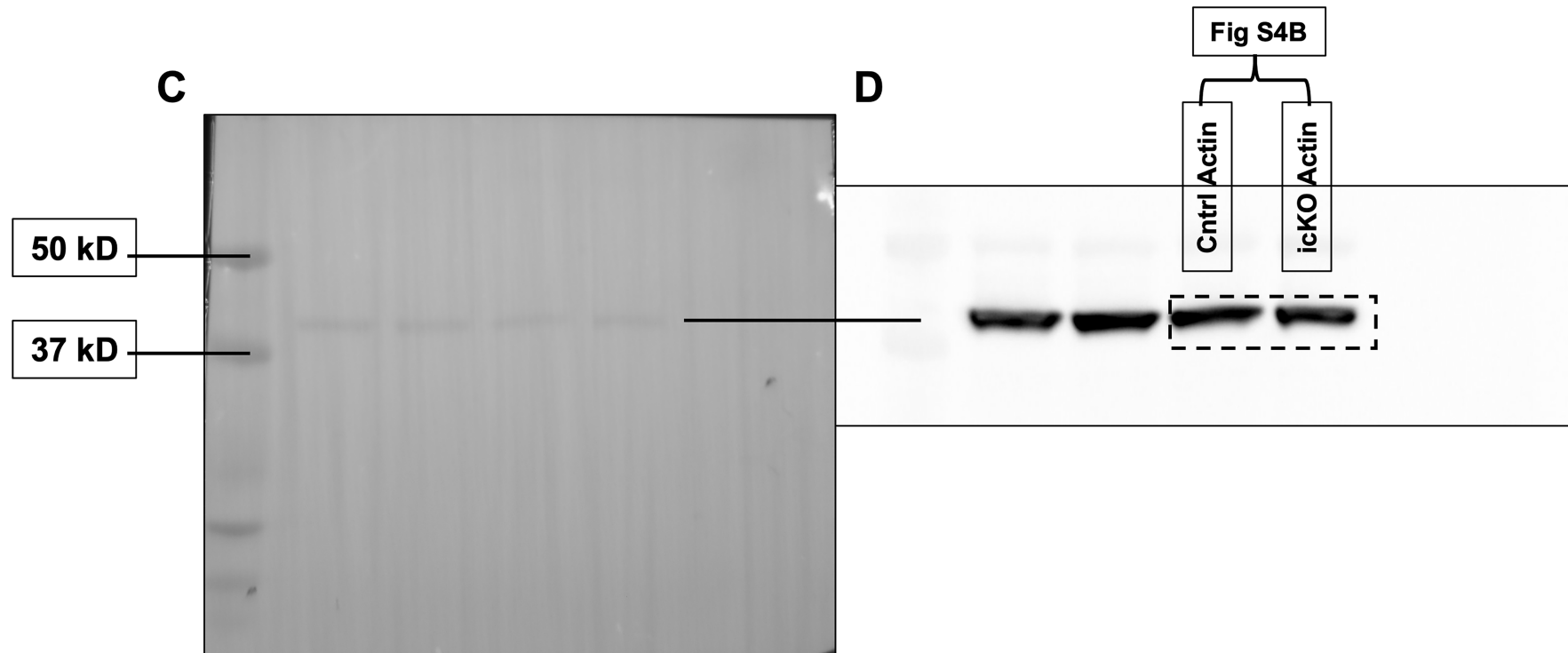

Supplement: SourceData FS4 — is the source file for Fig. S4. [file jcb_202311041_sourcedatafs4.pdf]

**Fig S5L**

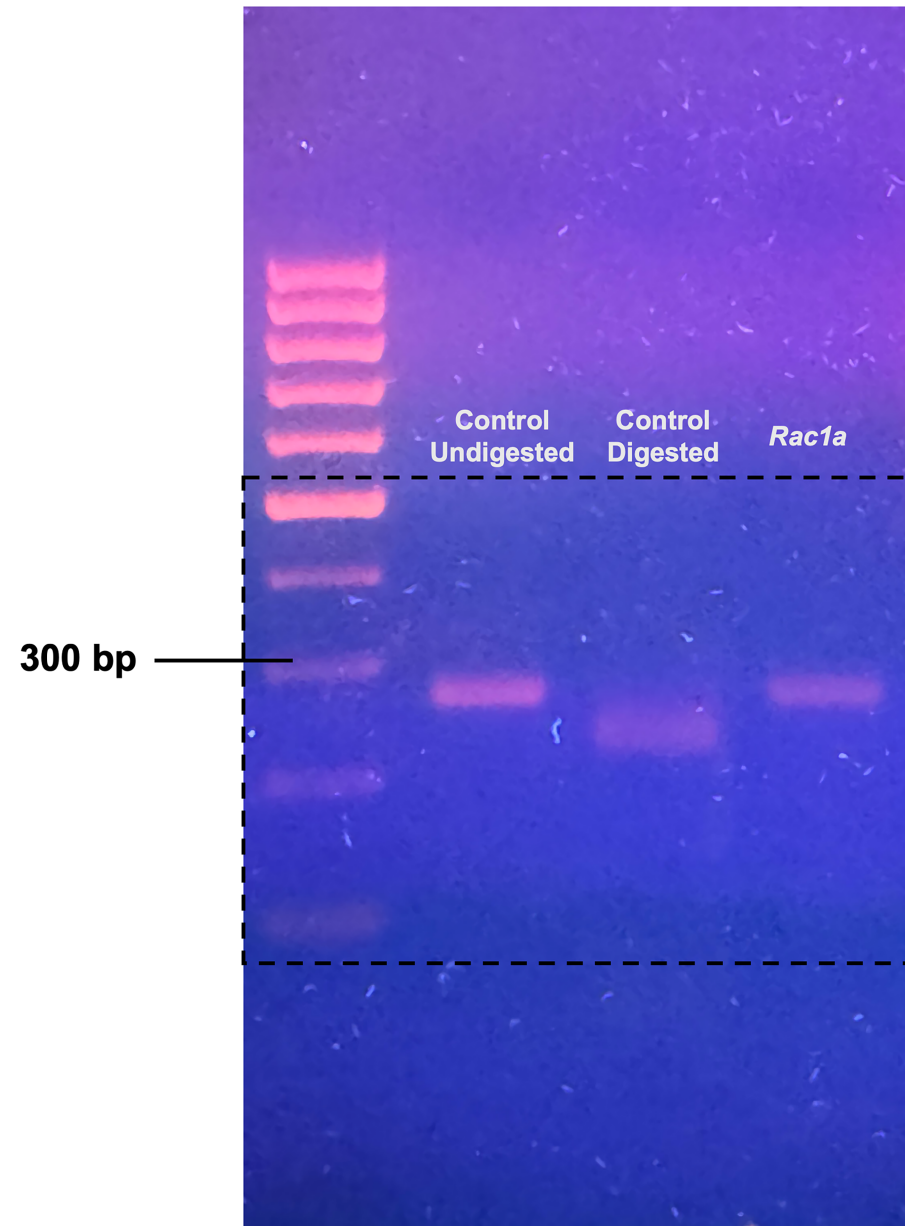

**Fig S5M**

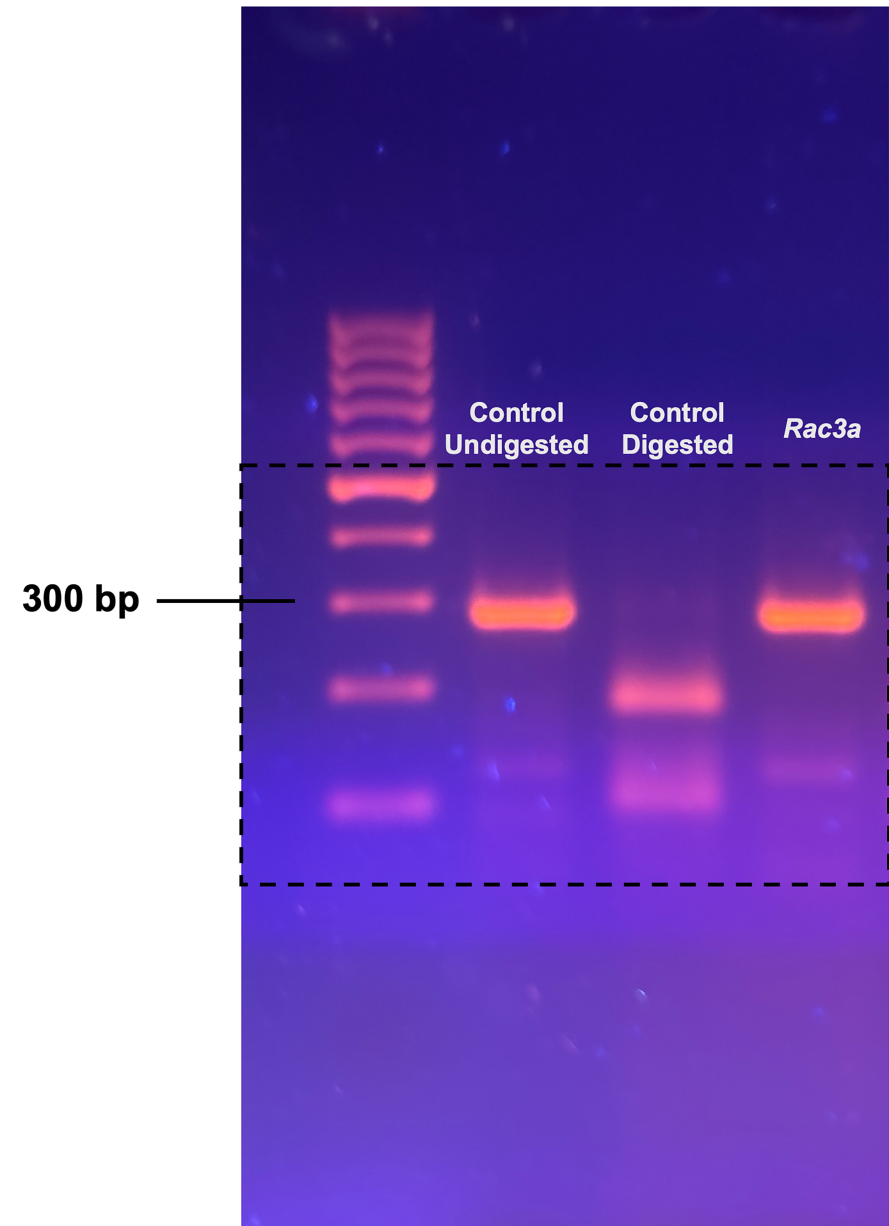

Supplement: SourceData FS5 — is the source file for Fig. S5. [file jcb_202311041_sourcedatafs5.pdf]
